# Influence of measurement method, measurement height, and sampling location on carbon dioxide and ammonia concentration in a conventional piglet nursery

**DOI:** 10.1016/j.vas.2026.100815

**Published:** 2026-08-14

**Authors:** Thies J. Nicolaisen, Nina Volkmann, Lena Brügge, Rolf Nathaus, Nicole Kemper, Jochen Schulz

**Affiliations:** a Institute for Animal Hygiene, Animal Welfare and Farm Animal Behaviour, University of Veterinary Medicine Hannover, Foundation, Hannover, Germany; b Institute of Animal Welfare and Animal Husbandry, Friedrich-Loeffler-Institut, Celle, Germany; c vetxperts Reken, Lindenweg 8, 48734 Reken, Germany

**Keywords:** Sampling point, Spatial distribution, Pig, Swine, Air quality, Harmful gases, Measurement technique

## Abstract

Low air quality and the presence of harmful gases such as ammonia can have detrimental effects on the health and welfare of pigs. Excessive concentrations of these gases are particularly common in negative pressure ventilated barns found in conventional husbandry systems. There are various devices and guidelines available for measuring harmful gases in such environments. In this study, ammonia and carbon dioxide were measured using three different devices. Measurements were taken across four production cycles in six pens at two different heights (0.6 m, within test pens; 1.5 m, representing human working height).

The measurement results for ammonia and carbon dioxide obtained with the three different methods differed significantly from one another. Comparison of the two measurement heights revealed no significant difference for ammonia. However, measurement height had a significant effect on carbon dioxide concentrations. Furthermore, the location of the sampling points (test pens) within the compartment significantly influenced the measured concentrations of both ammonia and carbon dioxide, indicating spatial variability.

When assessing air quality in a barn, it is advisable to use two different measurement systems for ammonia and carbon dioxide to ensure reliable results. Measuring ammonia does not necessarily have to be performed at animal height, as its vertical distribution was found to be uniform within the compartment. However, the location of the sampling points significantly influenced both ammonia and carbon dioxide concentrations. Therefore, measurements should be conducted at multiple locations within a compartment to capture spatial variability and to determine the most appropriate position for stationary sensors.

## 1 Introduction

Carbon dioxide and ammonia are continuously generated in livestock farming operations; for instance, carbon dioxide is produced both through animal respiration and decomposition of organic matter present in faeces and urine (Chmielowiec-Korzeniowska et al., 2022). In addition, ammonia—a volatile compound that exists as a gas at room temperature—is formed when the urea contained in animal urine is hydrolysed to ammonia by the enzyme urease, which is synthesized by microorganisms (Sigurdarson et al., 2018).

Managing carbon dioxide and ammonia in pig barns with mechanical ventilation can be challenging, as multiple factors contribute to varying concentrations of these gases within the barn. Therefore, management must always be considered within the context of overall barn climate regulation (e.g., balancing the prevention of excessive heat loss with the effective removal of harmful gases in piggeries under winter conditions). Insufficient removal of gases from the barn air results in increased concentrations in the animal area. This is problematic, as harmful gases have detrimental effects on the systemic health of pigs (Han et al., 2021; Xia et al., 2021, 4), particularly on respiratory health (Li et al., 2023; Michiels et al., 2015; Qin et al., 2022), as well as on performance (Cui et al., 2021) and the well-being and behaviour (Smith et al., 1996; Scollo et al., 2017). For this reason, recommendations for maximum concentrations of potentially harmful gases in pig barns exist in Germany (ammonia: 20 ppm [ppm]; carbon dioxide: 3000 ppm) (Anonymous, 2021) and in the European Union (ammonia: <10–15 ppm) (European Food Safety Authority (EFSA), 2022). However, in Germany, there are no legally binding maximum concentrations, nor established specifications for sampling points or measurement methods. Only recommendations for the measurement of harmful gases at federal level are available (LAVES, 2021).

Various technologies are available for measuring ammonia, including electrochemical gas sensors, photoacoustic gas sensors, laser absorption gas sensors, Fourier-transform infrared (FTIR) gas analyzers, and colorimetric gas sensors, which differ considerably regarding detection limits, measurement ranges, accuracy, cost, maintenance level and susceptibility to interfering factors (Aroh et al., 2026). Although electrochemical and colorimetric gas sensors generally exhibit lower accuracy than more advanced methods (e.g., FTIR), they are relatively inexpensive, user-friendly, and well suited for flexible application in routine use by farmers and veterinarians. The assessment of inter-device variability among low-cost measurement devices (Witt et al., 2024), as well as comparisons between a low-cost measurement system and a FTIR gas analyzer (Zhuang et al., 2019) under practical conditions in pig housings, has rarely been investigated.

Several studies have investigated the spatial distribution of noxious gases in pig barns; the recent study of Konapathri and Azinov (2024), based on computational fluid dynamic (CFD) simulations, demonstrated that ammonia distribution is highly heterogeneous and characterized by local hotspots, with ventilation and temperature as the primary controlling factors. A heterogeneous spatial distribution of ammonia and carbon dioxide was also observed in the CFD analysis by Tabase et al. (2020), which was primarily attributed to airflow patterns within the building. In contrast to the inhomogeneous ammonia distribution found both longitudinally and vertically by these studies, Jerez et al. (2010) found that gradients were primarily confined to the longitudinal direction, whereas no significant differences were observed along the vertical axis under practical barn conditions. The influence of the microenvironment – particularly temperature as part of a seasonal effect – on ammonia distribution was also highlighted in this study. This seasonal effect on ammonia distribution in a pig nursery was also demonstrated by Feng et al. (2022), who measured ammonia concentrations longitudinally along the aisle of a nursery compartment and observed significant spatial differences in winter that were not apparent in summer. Bahzani et al. (2013) conducted a large-scale study measuring indoor climate parameters across multiple pig production systems and, overall, found no significant differences between two sampling locations within the buildings.

Regarding measurement height, it is recommended to conduct measurements at animal level so that the recorded concentrations accurately reflect the pigs' actual exposure to harmful gases. Nonetheless, measuring at animal height is only feasible at certain times and/or when the measuring equipment is adequately shielded from the pigs, as their exploratory behaviour may interfere with the measurement or damage the equipment. Recent technological advances have enabled the automatic and continuous measurement of harmful gases in barns using specific sensors. However, measurements with these systems are often performed above animal height for the aforementioned reasons.

Therefore, this study adopted a practice-oriented approach, focussing on the assessment of inter-device variability among three measurements systems for ammonia and carbon dioxide specifically, two hand-held devices (an electrochemical and a colorimetric gas sensors) used for spot measurements and a device for continuous long-term measurement - suitable for cost-effective and practical use by farmers and veterinarians under on-farm conditions. Measurements were conducted at two different heights and at different sampling locations within one compartment of a piglet nursery. The used gas detection tubes are well described and widely used in agricultural ammonia measurements (Bonizzi et al., 2022; Hahne, 2024; Ni & Heber, 2001; Witt et al., 2024). Furthermore, the tubes and the electronic hand-held device are suggested as mobile devices to control ammonia, carbon dioxide and hydrogen sulphide in livestock buildings by the Lower Saxony State Office for Consumer Protection and Food Safety – LAVES (LAVES, 2021). The electronic hand-held device used is a certified system for the field of occupational safety and is also applied in livestock research for the measurement of ammonia concentrations (Chen et al., 2020; Such et al., 2023). The continuously monitoring system that measures carbon dioxide, and ammonia has not yet been used in a livestock building.

## 2 Material and methods

### 2.1 Animals and housing conditions

The study was conducted over four production cycles (all-in-all-out system) in a conventional piglet nursery in northwest Germany between January 2021 and August 2021. Production cycles lasted 27 days in the first run and 50 days in each of the three subsequent runs. Animal weights delivered by the farmer were 13 kg – 24 kg, 8 kg – 33 kg, 8 kg – 36 kg and 8 kg – 35 kg. The facility, constructed symmetrically in 2019, consisted of a central aisle flanked by seven compartments on each side. Each compartment measured approximately 30 m × 7 m and contained 20 pens of about 3 m × 3 m each. Twenty-five piglets were housed in each pen, resulting in a total of 500 piglets per compartment. The livestock owner reported an average loss of 3% in livestock numbers. The mechanical ventilation system operated under negative pressure: fresh air was supplied to the compartments from the central aisle through a perforated ceiling, while exhaust air was removed via a fan located centrally in each compartment (Co. Menken und Drees GmbH, Coesfeld, Germany). A twin-pipe heating system was installed in the compartments. Feed was delivered through automated liquid feeding stations. Manure channels extended along the entire length of each compartment, measuring 0.5 m in depth and 2.0 m in width, equipped with slurry slides and flushing pipes. Cleaning and disinfection of the compartments were performed after each experimental batch.

### 2.2 Data collection

**Fig. 1:**
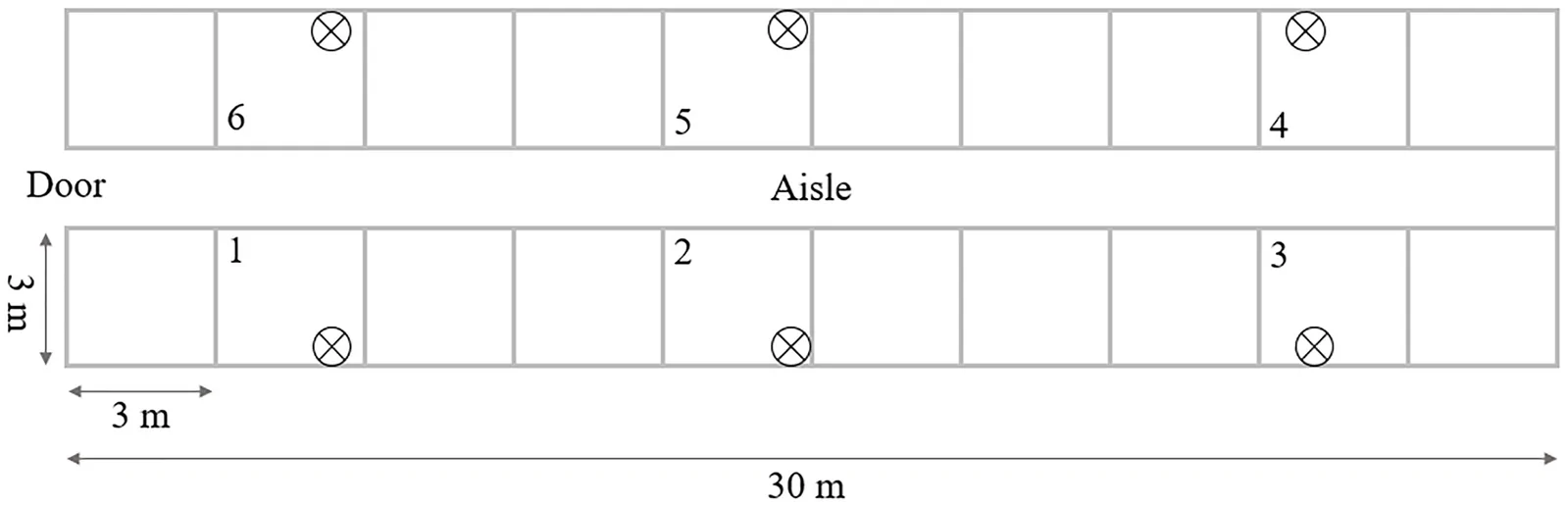
Experimental compartment of the piglet nursery. Numbers (1–6) mark the test pens. The sign cross in a circle shows the sampling points in the test pens for all three measuring methods.

Data collection was conducted in a single compartment located at the centre of the barn. The selected compartment was situated on the northwest side of the building. Measurements were taken in all four experimental batches at one time point per week (a total of 31 time points between January and August 2021). Six sampling points (test pens) were designated within the compartment. For this purpose, the compartment was divided into thirds, with two measurement points established in opposite pens within each third (Fig. 1). At each of the six sampling points, measurements were performed at two different heights: 0.6 m and 1.5 m. A measurement height of 0.6 m was chosen to compare manual measurements with fixed sensor systems in the pens, while 1.5 m corresponds to the typical working height for humans. The following parameters were recorded: air temperature, relative humidity, air pressure, air velocity (horizontal and vertical), carbon dioxide concentration, and ammonia concentration.

Air temperature, relative humidity, and air pressure were measured using a digital hand-held device (PCE–THB 40, PCE Prüfgeräte Deutschland GmbH, Meschede, Germany). Both vertical and horizontal air velocities were assessed using a hot-wire anemometer (Testo 405-V1, Testo SE & Co. KGaA, Titisee-Neustadt, Germany).

Ammonia and carbon dioxide concentrations were measured at both heights and at all measurement points using two to three different methods: a digital handheld multi-gas measuring device (DHH), a handheld pump measuring device for ampoule tubes (PMD), and a continuously measuring sensor system (CMS). All manual measurements were taken between 8.30 am and 1.30 pm. The exact times are listed in Table S1.

1) The DHH (Dräger X-am 5600, Model MQG 01, Dräger Safety AG & Co. KGaA, Lübeck, Germany) was equipped with an electrochemical sensor for ammonia (DrägerSensor® XXS NH₃, measuring range: 0–300 ppm; precision: ±3%; resolution: 1 ppm) and an infrared sensor for carbon dioxide (DrägerSensor® IR CO₂ ES, measuring range: 0–5% by volume; accuracy: 0.01% by volume; resolution: 50 ppm). The device was calibrated by the manufacturer prior to data collection and in July 2021. No measurements were taken during that week. According to the user manual, the device was switched on in the outer air to perform a zero calibration before the measurements in the barn.
2) The PMD (Accuro® pump, Dräger Safety AG & Co. KGaA, Lübeck, Germany) was used in combination with the corresponding tube systems (DrägerTubes® Ammonia 5/a: measuring range: 5–70 ppm, standard deviation: ±10–15%, estimated resolution: 1 ppm; DrägerTubes® Carbon Dioxide 0.1%/a: measuring range: 0.1–1.2% by volume, standard deviation: 5–10% by volume, estimated resolution: 0.025%). A defined volume of air was drawn by a specified number of pump strokes through a glass ampoule containing a substrate that reacts with the target gas, resulting in a colour change. In this single-use system, the measured value was read from a scale on the ampoule. Measurements with gas tubes were conducted every two weeks in two randomly selected pens (one pen always in the middle third of the compartment; the second pen in either the inner or outer third), and both measurement heights were utilized in the selected pens.
3) The CMS was provided by Clevabit GmbH (Emsdetten, Germany) and included sensors for carbon dioxide (Sensirion SCD30; Sensirion AG, Stäfa, Switzerland; measuring range: 0–40,000 ppm; accuracy: ±30 ppm; resolution: 1 ppm) and ammonia (ECsense EC4-NH₃, ECsense GmbH, Hohenschäftlarn, Germany; measuring range: 0–100 ppm; accuracy ±5%; resolution: 0.1 ppm). Two sensor boxes were installed in each test pen at 0.6 m and 1.5 m. Calibration of the ammonia sensors was performed four times during the study by the manufacturer, using test gas containing 50 ppm and 100 ppm ammonia (All-in-Gas GmbH, Starnberg, Germany). To compare the concentrations of manual measurements with the CMS system, the times of measurements were recorded precisely so that the data could later be compared with the data received from the automated system. The CMS recorded the readings every minute. The lower box (0.6 m height) was protected by a stainless-steel cage constructed with a sufficiently large, slotted area to ensure measurement accuracy while preventing pig interference. The cage was made of 4 mm thick stainless steel, with dimensions of 600 mm in height and 280 mm in width. The slot width was 10 mm. The total surface area of the cage was 193,000 mm², of which 128,000 mm² was slotted. Additionally, 150,000 mm² of open area was provided at the top and bottom edges of the cages.

The values for CO₂ were measured as percent by volume and subsequently converted to parts per million (ppm), using the relationship: 1 ppm = 0.0001%. The measurements of NH₃ and CO₂ obtained with the CMS were automatically corrected using internal air pressure measurements. Values for NH₃ and CO₂ obtained with the PMD were corrected using the results from simultaneous air pressure measurements, in accordance with the following Eq. (1) provided in the manufacturer’s instructions:

(1) $$corrected\;gas\;value=\text{measured}\;\text{gas}\;\text{value}*\left(\frac{1013}{\text{measured}\;\text{air}\;\text{pressure}}\right)$$

### 2.3 Statistical analysis

The results of this study were processed with Microsoft Excel (Microsoft Corporation, Redmond, WA, USA) and statistically analysed with SAS version 9.4 (SAS Institute Inc., Cary, NC, USA).

A generalized mixed model (GLIMMIX procedure) was used to analyse the statistical effects on carbon dioxide and ammonia concentration. Model assumptions were checked visually using Q-Q plots for a normal error distribution and homoscedasticity of residuals. Manual, backward, stepwise elimination was used to remove variables with the largest p-value until only variables with a p-value < 0.05 or those that improved the model fit were retained in the model. Model fit was assessed by minimization of Akaike-Information-Criterion (AIC) and Bayesian-Information-Criterion (BIC) values. The best fitting model for carbon dioxide concentration included the random effects of the interaction of date and batch and the fixed effects of measurement height (0.6 m and 1.5 m), air temperature, measuring method (method 1: digital hand-held multi-gas measuring device, method 2: pump measuring device for ampoule tubes, method 3: continuous measuring sensor system), air velocity (vertical), relative air humidity, batch (1–6), and date. A further generalized mixed model for ammonia concentration included the interaction of date and batch as random effect and the fixed effects of measurement height (0.6 m and 1.5 m), air temperature, measuring method (method 1: digital hand-held multi-gas measuring device, method 2: pump measuring device for ampoule tubes, method 3: continuous measuring sensor system), air velocity (vertical), batch (1–6), and date. Post-hoc pairwise comparisons were conducted using Tukey-Kramer tests. For all statistical analyses, p values of < 0.05 were considered statistically significant.

### 2.4 CO_2_ mass balance method

There was no opportunity to measure the ventilation rates using artificially injected tracer gas methods or fan wheel anemometers. A recommended model for calculation of the ventilation flow, based on carbon dioxide concentrations and corrected by the relative animal activity factor for weaners, the temperature correction factor and an additional CO_2_ charge for slurry, was used to estimate air flow rates in the investigated compartment (Pedersen, 2008; CIGR report, 2002). A fixed background value of 420 ppm was used in the model, as it approximates the mean atmospheric CO₂ background concentration in Germany during the study period (Umweltbundesamt, 2024). Average CO_2_ concentrations measured with the DHH device have been incorporated into the model (Eq. (2)). These concentrations and other factors and information used for the model are listed in Table S1.

(2) $$V=\frac{n*\left[\Phi_{m}*\left(1+2.2*\left(1-k\right)\right)\right]*0.000185*a*\;k_{T}*1.1\;}{C_{i}-C_{o}}$$

where.

V: Ventilation flow rate [m³/h]. n: Number of animals in the building.

Φ_m_: Maintenance heat production according to CIGR (5.09 · m^0.75^) [W]. m: Average live body mass [kg]. k: Factor for energy retention (0.47 + 0.003 · m) [dimensionless].

0.000185: CO_2_ production coefficient according to Pedersen [m³/(h·W)]. a: Relative animal activity factor [dimensionless].

*k_T_:* Temperature correction factor for T > 20 °C (1 - 0.012 · (T-20)).

1.1: Correction factor for additional CO_2_ sources from manure.

*C_i_, C_o_*: concentration of indoor and outdoor air [ppm].

A feeding level of n = 3.2 times the maintenance energy requirement was assumed for weaned pigs (7.5–30 kg) according to (CIGR report, 2002). Further, we set the animal activity factor to 1.2 according to the CIGR report, (2002) because most of the measurements were conducted in the late morning or at midday. Air flow rates were calculated in Microsoft® Excel® for Microsoft 365 (Version 2602, Microsoft Corporation). The results are also shown in Table S1.

## 3 Results

Descriptive statistics for the barn climate parameters—air temperature, relative air humidity, vertical air velocity, and horizontal air velocity—as well as for the three different methods used to measure carbon dioxide and ammonia, are presented in Table 1, Table 2.

**Table 1:** Descriptive statistics for the barn climate parameters of ammonia and carbon dioxide (in parts per million) at 0.6 m height and 1.5 m height. PMD = handheld pump measuring device; DHH: digital handheld multi-gas measuring device; CMS = continuously measuring sensor system.

Climate parameter		Measuring height	Number of measurements	Mean	Minimum	Maximum	Standard deviation
Carbon dioxide	PMD	0.6 m	34	3291	1996	5018	904.6
		1.5 m	34	3316	2003	5269	943.0
	DHH	0.6 m	186	3034	1300	5200	872.5
		1.5 m	186	3187	1250	5500	971.2
	CMS	0.6 m	177	2738	1089	5052	912.0
		1.5 m	175	2753	872	4810	929.6
Ammonia	PMD	0.6 m	34	27	7	60	14.6
		1.5 m	34	26	7	60	14.5
	DHH	0.6 m	186	27	5	65	13.8
		1.5 m	186	27	4	67	14.3
	CMS	0.6 m	175	33	0	55	9.8
		1.5 m	173	34	0	59	9.3

**Table 2:** Descriptive statistics for the barn climate parameters air temperature (in Celsius = °C), relative air humidity (in percent = %), and air pressure (in hectopascal = hPa) at 0.6 m height and 1.5 m height.

Climate parameter	Measuring height	Number of measurements	Mean	Minimum	Maximum	Standard deviation
Air temperature ( °C)	0.6 m	186	29.4	25.3	32.6	1.3
	1.5 m	186	29.5	25.4	32.6	1.3
Relative air humidity (%)	0.6 m	186	69.7	45.8	93.2	10.8
	1.5 m	186	69.6	45.1	93.2	10.8
Air pressure (hPa)	0.6 m	186	1008.3	985.0	1027.3	9.4
	1.5 m	186	1008.3	985.2	1027.2	9.4
Air velocity (m s^-1^)	vertical 0.6 m	186	0.06	0	0.21	0.05
	vertical 1.5 m	186	0.08	0	0.36	0.07
	horizontal 0.6 m	186	0.03	0	0.22	0.03
	horizontal 1.5 m	186	0.04	0	0.18	0.04

**Fig. 2:**
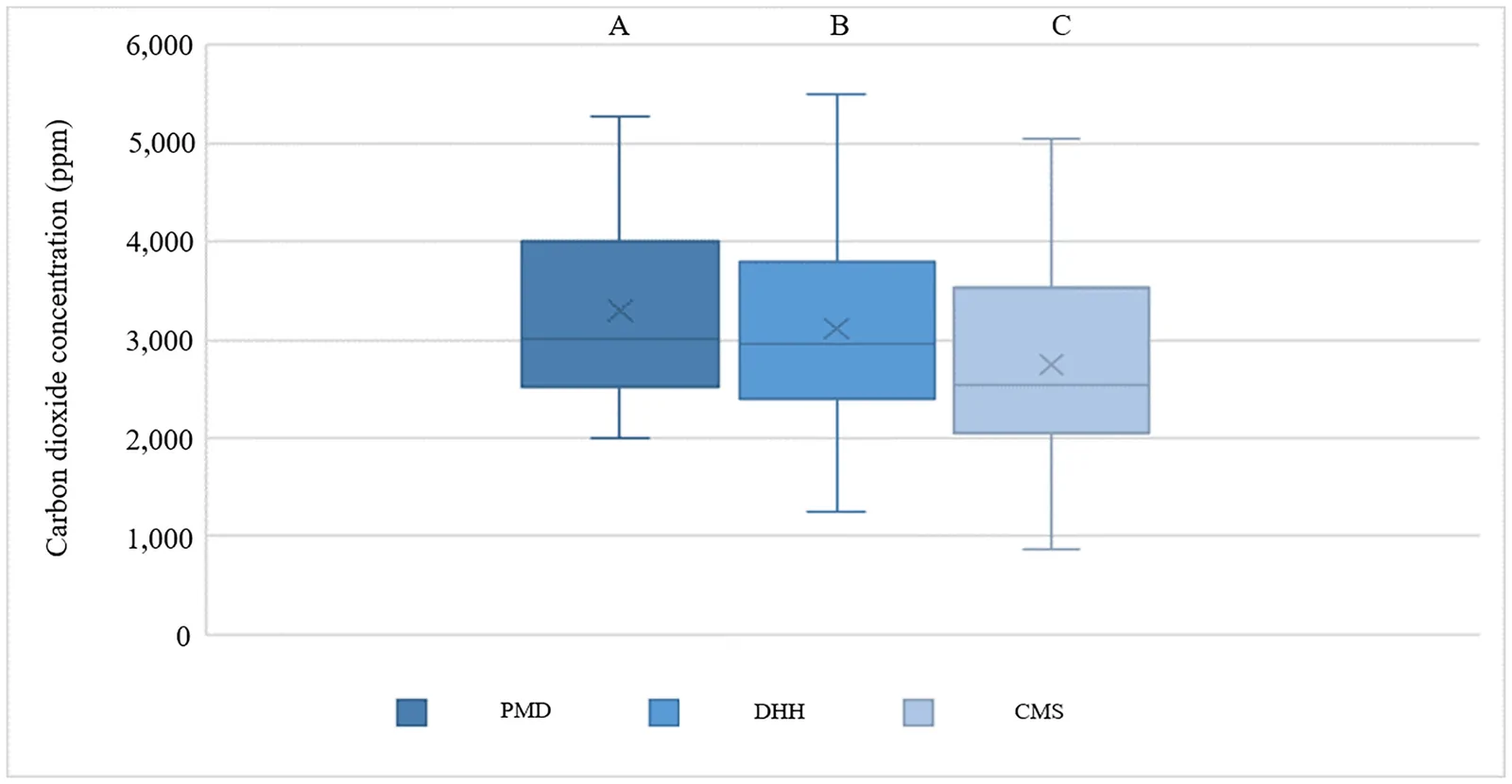
Box-plot diagram of the measured carbon dioxide concentrations with the three different devices (PMD = handheld pump measuring device; DHH: digital handheld multi-gas measuring device; CMS = continuously measuring sensor system.). The filled-in areas represent the two middle quartiles. The line in the middle quartiles represents the median, the cross represents the mean. The lower whisker represents the first quartile; the upper whisker represents the fourth quartile. Different letters (A, B, C) mark significant differences between the measuring systems (*p* < 0.05).

The generalized mixed model demonstrated that carbon dioxide concentration was significantly influenced by measuring height (*p* = 0.0278), air temperature (*p* = 0.0117), measuring method (*p* < 0.01), relative air humidity (*p* < 0.01), pen (*p* = 0.0495), and date (*p* < 0.01). No significant effect was observed for vertical air velocity (*p* > 0.05). Pairwise comparisons indicated significant differences in carbon dioxide concentrations among all three measurement methods: DHH vs. PMD (*p* = 0.0011), DHH vs. CMS (*p* < 0.0001), and PMD vs. CMS (*p* < 0.0001). (Fig. 2). Although the generalized model identified an effect of pen on carbon dioxide concentration, pairwise comparisons did not reveal significant differences between individual pens.

The second generalized statistical model on ammonia concentration identified significant effects of the fixed factors measuring method (*p* < 0.0001), measurement pen (*p* < 0.0001), and date (*p* < 0.0001). In contrast, measurement height (*p* = 0.5698), air temperature (*p* = 0.5747), and vertical air velocity (*p* = 0.0947) did not have statistical significant effects.

**Fig. 3:**
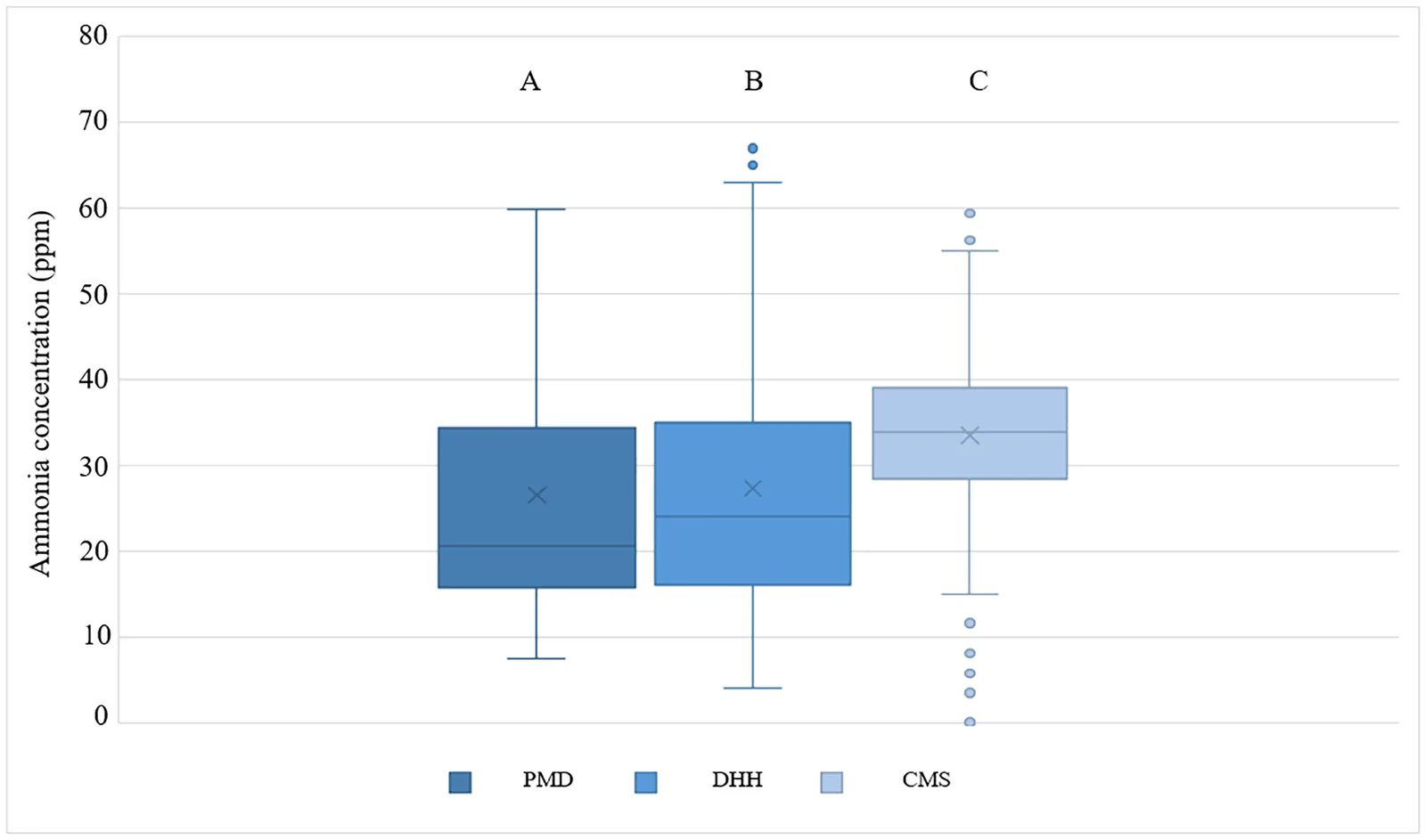
Box-plot diagram of the measured ammonia concentrations with the three different devices PMD = handheld pump measuring device; DHH: digital handheld multi-gas measuring device; CMS = continuously measuring sensor system. The filled-in areas represent the two middle quartiles. The line in the middle quartiles represents the median, the cross represents the mean. The lower whisker represents the first quartile; the upper whisker represents the fourth quartile. Points outside the whiskers mark outliers. Different letters (A, B, C) mark significant differences between the measuring systems (*p* < 0.05).

Pairwise comparisons revealed significant differences among all three measuring methods: DHH vs. PMD (*p* = 0.0475), DHH vs. CMS (*p* < 0.0001), and PMD vs. CMS (*p* < 0.0001). The measured concentrations are presented in Fig. 3. No significant differences in ammonia concentration were observed between the two measurement heights (0.6 m and 1.5 m; *p* = 0.5698).

Multiple significant differences in ammonia concentration were detected between individual measurement pens (pen 1 vs. pen 2: *p* < 0.0001; pen 1 vs. pen 4: *p* = 0.0434; pen 1 vs. pen 5: *p* < 0.0001; pen 2 vs. pen 3: *p* < 0.0001; pen 2 vs. pen 6: *p* < 0.0001; pen 3 vs. pen 4: *p* = 0.0002; pen 3 vs. pen 5: *p* < 0.0001; pen 4 vs. pen 6: *p* = 0.0165; pen 5 vs. pen 6: *p* < 0.0001; all other pen comparisons: *p* > 0.05) .

**Fig. 4:**
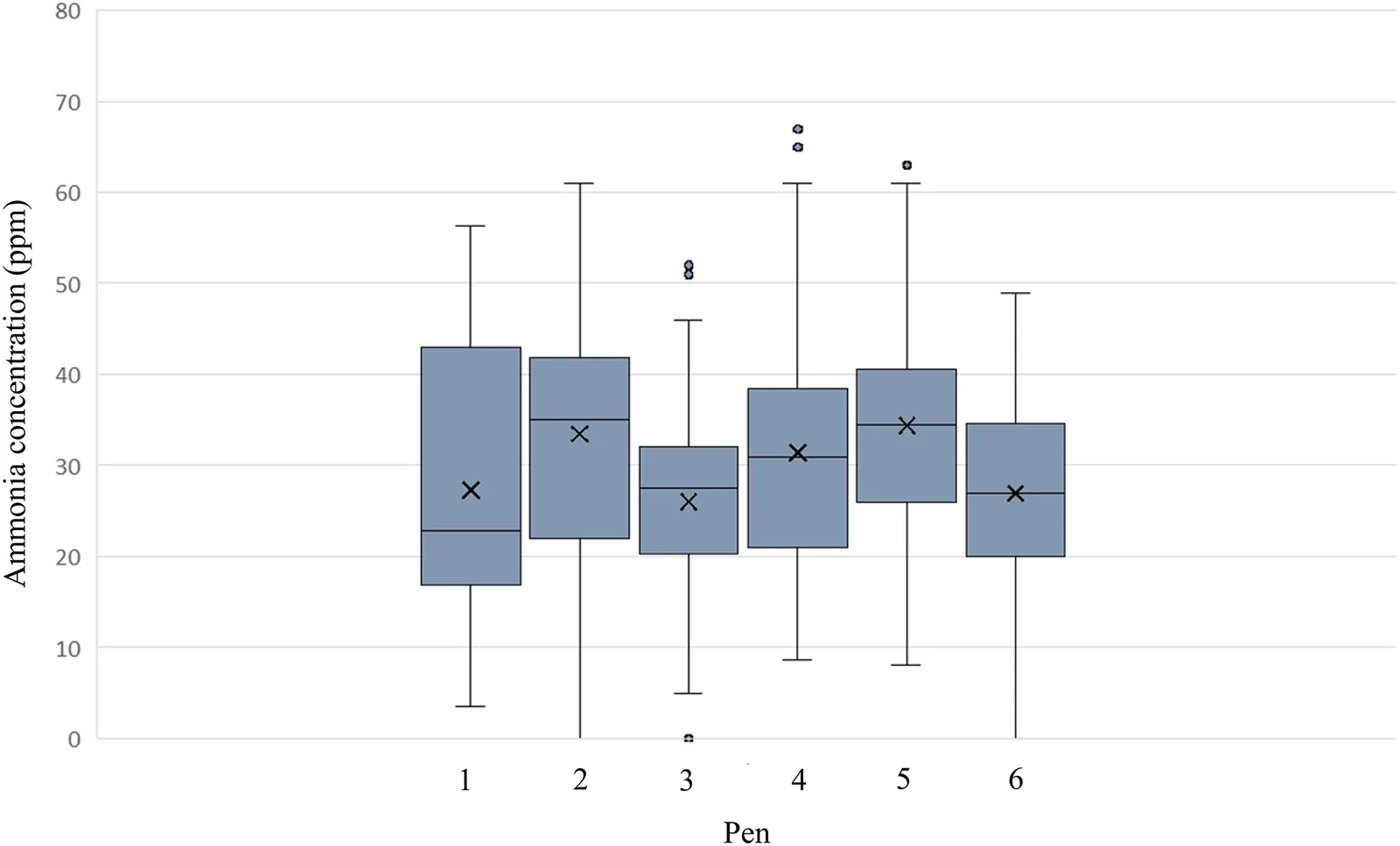
Box-plot diagram of the measured ammonia concentration (measurements from all devices included, in parts per million = ppm) in the six measurement locations (test pens; 1–6). The filled-in areas represent the two middle quartiles. The line in the middle quartiles represents the median, the cross represents the mean. The lower whisker represents the first quartile; the upper whisker represents the fourth quartile.

**Fig. 5:**
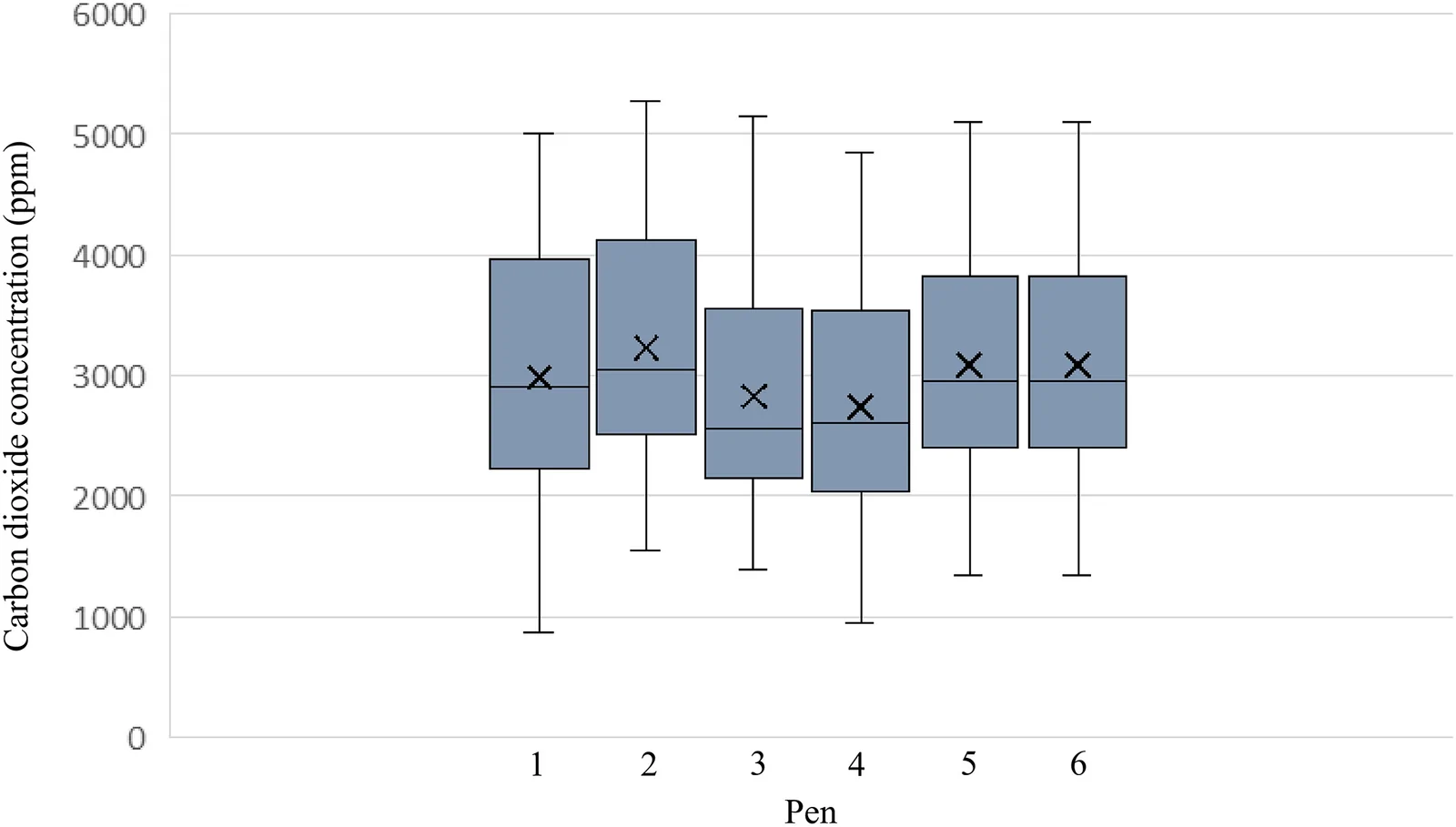
Box-plot diagram of the measured carbon dioxide concentration (measurements from all devices included, parts per million = ppm) in the six measurement locations (test pens; 1–6). The filled-in areas represent the two middle quartiles. The line in the middle quartiles represents the median, the cross represents the mean. The lower whisker represents the first quartile; the upper whisker represents the fourth quartile.

Figure 4 and Figure 5 present an overview of the measurement results for ammonia and carbon dioxide obtained (measurement results from all devices included) in the six experimental pens.

**Fig. 6:**
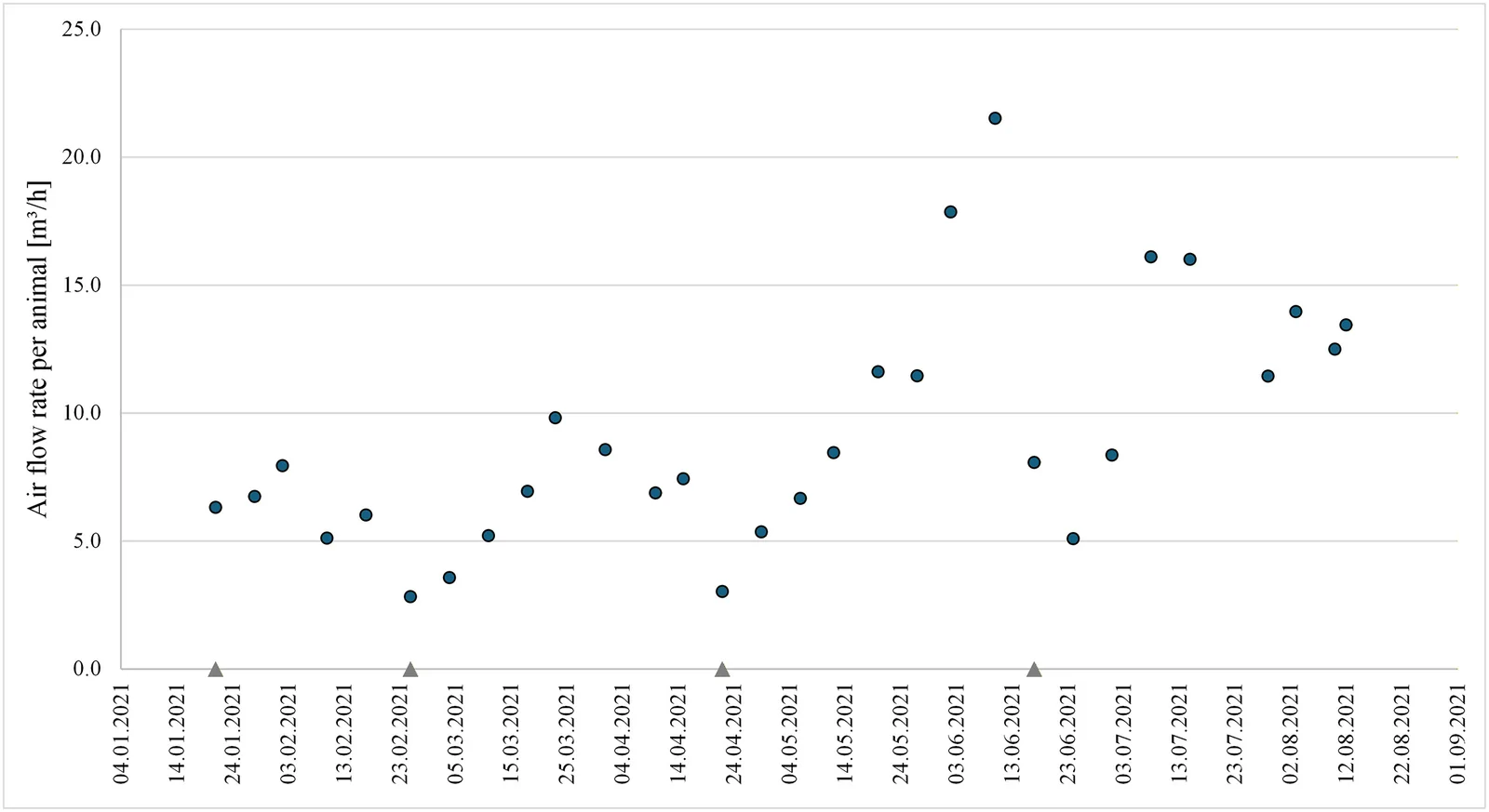
Air flow rates calculated by the CO_2_ mass balance method. Black dots (●) show the air flow rates per animal during the measurements within the compartment. Triangles (▲) on the time axis mark the beginning of the four production cycles.

Results of the measurements in the compartment showed partly high carbon dioxide and ammonia concentrations. To estimate if this could have been an effect of insufficient ventilation, a model for calculation air flow rates based on indoor carbon dioxide concentrations was used. The air flow rates per animal ranged between 3 and 22 m³/h (Fig. 6). Higher air flow rates (> 10 m³/h) were observed during the warm season between late May and mid-August. The values fluctuated within a production cycle. The air flow rates increased at the beginning of the production cycles. In three cases the air flow rates decreased towards the end of the growing cycles. The calculated air flow rates were compared to recommended air flow rates in the discussion section.

## 4 Discussion

The objective of this study was to compare different methods for measuring ammonia and carbon dioxide concentrations under practical conditions in a piglet nursery unit and to assess the influence of both measurement position and height on the measurement outcomes.

The statistical models revealed significant influences of the measurement methods on carbon dioxide and ammonia concentrations. Plots and p-values from pairwise tests showed that ammonia and carbon dioxide concentrations measured by the CMS device exhibited greater deviations compared to PMD and DHH devices. To our knowledge, the CMS system has not previously been tested in livestock buildings, whereas, as mentioned in the introduction, the other two devices are established systems used for gas detection in such environments. Hahne (2024), for example, reported a coefficient of variation of 15% for the ammonia test tubes (PMD device) in pig barns. The DHH was evaluated and showed a coefficient of variation of 9% at room temperature in air with 20 ppm ammonia (Lee et al., 2026). The CMS appears to significantly overestimate ammonia concentrations and underestimate carbon dioxide concentrations. The reasons for this remain unknown. The precision of electrochemical sensors in livestock buildings can be affected by various factors such as other pollutants or high humidity. As suggested by Lee et al. (2026), more comprehensive laboratory studies may help improve the precision of sensors for gas detection in livestock buildings.

The study was conducted in a commercial piglet nursery that had been constructed in 2019 and featured a typical ventilation system. The compartment was divided into 20 pens and stocked with 25 weaners per pen. In the investigated compartment of the present study, samples were taken from three pens on each side at two heights (0.6 m and 1.5 m). The experimental setup and the number of samples enabled the detection of both a pen effect and a height effect in the statistical model. For instance, pen 2 and pen 5 in Fig. 4, Fig. 5, both located in the middle section of the compartment, showed the highest average concentrations for ammonia and carbon dioxide. Air velocities were generally low in all pens and showed no significant effect on gas concentrations. The reasons for the higher concentrations in the middle of the compartment remain unknown. Spatial variability has been reported in previous studies (Sturm et al., 2021). Factors such as manure management, animal activity, airflow, and the position of the pens relative to the exhaust point may have influenced the measured carbon dioxide and ammonia concentrations (Deb et al., 2025, 2023; Peters et al., 2012). However, it is suggested that achieving uniform air quality within a compartment is feasible and should be pursued to ensure consistently favorable conditions for all animals (Banhazi, 2013).

Carbon dioxide measurements obtained at 1.5 m showed higher values than those recorded at 0.6 m. The generalized mixed model showed a significant height effect on carbon dioxide concentration, although the maximum difference in the measured mean concentration was approximately 100 ppm. Carbon dioxide is primarily generated through pigs’ respiration at animal height (Ni et al., 1999a). For instance, one study found higher concentrations in the breathing zone of pigs compared to higher sensor positions (Müller et al., 2006). The vertical spatial differences of carbon dioxide concentration observed in our study are likely the result of insufficient turbulent mixing of the compartment air. Turbulence caused by animal activity during the measurements may also have influenced carbon dioxide concentrations at the lower position. The lower height in the present study was 0.6 m, which is above the suggested measurement heights for pigs in other studies (Li et al., 2025; Müller et al., 2006). We cannot exclude the possibility that carbon dioxide and ammonia measurements near ground level would have shown different results. In terms of occupational safety, 1.5 m should be the preferred height. When the focus is on animal health, measurements should be taken at animal height. In contrast to carbon dioxide, no vertical spatial variation in ammonia concentration was detected in the present study, indicating that ammonia concentrations were largely independent of measurement height. Comparable results were reported by Jerez et al. (2010)., who likewise found no significant differences between measurements obtained at heights of 0.8 m and 1.6 m. In contrast, computational fluid dynamics (CFD)-based studies have shown that vertical ammonia concentration gradients may occur (Konapathri & Azinov, 2024; Tabase et al., 2020)

The spatial differences in gas distribution observed in the present study are consistent with findings from previous studies conducted in livestock buildings and are likely attributable to microclimatic conditions within the barn. .Influencing factors for a difference in spatial distribution of gases include temperature and season, ventilation and air flow (Feng et al., 2022; Jerez et al., 2010; Konapathri & Azinov, 2024), flooring type (Aarnink et al., 1997), defecation areas within the pen, slurry pit management, airflow dynamics (Granier et al., 1996), and proximity to air inlets or exhaust points. These factors may change over time, affecting gas distribution. Therefore, relying on a single stationary sensor may be insufficient unless its placement has been optimized. If only one sensor is used, it should be placed in the area with the poorest air quality. In practice, multiple measurement locations are recommended, which can be effectively achieved using hand-held devices (Ni & Heber, 2001). While such devices are flexible, spot measurements are time-consuming, and gas concentrations vary with time of day and season. Therefore, repeated measurements are necessary to capture diurnal patterns (Ni & Heber, 2001). Additionally, studies such as Peng et al. (2023) suggest that artificial intelligence can help predict threshold exceedances, enabling proactive interventions.

Our results revealed that the recommended threshold values for both ammonia and carbon dioxide (EFSA, 2022) were frequently exceeded. Thus, proper ventilation remains an issue in commercial piglet nurseries. Especially at the beginning of a growing cycle, piglets require high temperatures. Farmers usually do not measure carbon dioxide or ammonia concentrations and often reduce ventilation rates, particularly at the beginning of the cycle and during cold weather, to maintain high barn temperatures. In the present study, temperatures in the compartment ranged between 27 °C and 32 °C (Table S1). The calculated airflow rates per animal (Fig. 6) confirmed relatively low values during colder seasons and at the beginning of growing cycles. In summer, airflow rates per animal increased toward the end of growing cycles by a factor of 2 to 3. This may explain the significant date effect on the measured carbon dioxide and ammonia concentrations. For instance, the average concentrations for carbon dioxide and ammonia calculated from Table S1 during the second growing cycle (end of February to mid-April) were 3860 ppm and 27 ppm, respectively. In contrast, during the growing cycle from July to mid-August, carbon dioxide averaged 2487 ppm and ammonia 21 ppm. The carbon dioxide mass balance method used provides a reasonably accurate estimate of ventilation rates in mechanically ventilated livestock buildings (Mosquera et al., 2012). An animal activity factor was included in the model because high activity levels were expected during measurements. The calculated airflow rates per animal were close to or below the recommended rates for weaners specified in DIN 18910:2017–08 (2017). Higher airflow rates would likely reduce average gas concentrations in the compartment; however, optimal temperatures for piglet growth must also be maintained. This finding indicates that effective removal of harmful gases in enclosed livestock facilities remains a challenge.

Although this study used spot measurements to compare sensors, the repeated detection of high ammonia levels indicated that recommended thresholds were exceeded. In addition, especially in the middle of the compartment (pen 2 and 5, Fig. 4) the median ammonia concentrations were > 30 ppm.

The formation and concentration of ammonia within a barn are influenced by various factors that can contribute to elevated concentrations. Among these, the type and management of the slurry system are considered to have the greatest impact (Monterny, 1996). Elevated ambient temperatures, as observed in the present study, may increase floor contamination with faeces due to altered elimination behaviour in pigs, leading to higher ammonia emissions (Ni et al., 1999b). Additionally, flooring characteristics, such as the extent of perforation, also affect emission rates (Aarnink et al., 1996, 1997; Sun et al., 2008; Ye et al., 2009). For example, partially slatted floors generally results in lower emissions than fully slatted floors, while deep litter systems tend to produce higher ammonia emissions compared to slatted floors (Philippe et al., 2007).

It remains unclear whether the elevated concentrations observed in our study adversely affected the pigs. In general, the detrimental effects of ammonia on pig health are well documented. These include systemic organ damage (Han et al., 2021; Xia et al., 2021), particularly affecting the respiratory tract (Li et al., 2023; Michiels et al., 2015; Qin et al., 2022; Urbain et al., 1994). Higher ammonia exposure is associated with increased risk of *Mycoplasma hyopneumoniae* infection, a greater incidence of pulmonary lesions (Michiels et al., 2015; Witt et al., 2024), and pleurisy (Donham, 1991; Michiels et al., 2015). Furthermore, elevated ammonia concentrations negatively affect animal welfare, behavioural patterns and growth performance. Choice experiments have shown that pigs exhibit a preference for compartments with lower ammonia concentrations, indicating clear aversion to ammonia exposure (Smith et al., 1996; Whates et al., 2002). Increased ammonia concentrations have also been shown to alter active behaviour and reduce growth performance in pigs (Sturm et al., 2021).

After data analysis of the present study, the farm veterinarian was informed and may have discussed mitigation strategies with the farmer. However, continuous monitoring systems are necessary to capture diurnal fluctuations, detect unexpected variations, provide timely warnings when thresholds are exceeded and enable a more accurate assessment of the animals’ actual exposure to noxious gases.

Diurnal variations have been reported, with elevated gas concentrations at night due to decreased ventilation rates (Rodriguez et al., 2020). Regarding seasonal differences, previous studies have shown that harmful gas concentrations, such as ammonia, are typically higher in winter than in summer (Aarnink et al., 1995; Blunden et al., 2008). This can be explained by lower air exchange rates in winter due to reduced ventilation for heat conservation, whereas higher air exchange and increased emissions occur during warmer conditions (Harper et al., 2004). Higher airflow rates in summer were also observed in this study. However, since piglet nurseries require higher temperatures, this seasonal effect is expected to be less pronounced than in fattening pig units.

## 5 Conclusion

The results of this study clearly demonstrated significant inter-device variability, with the three measurement systems differing significantly from each other in both ammonia and carbon dioxide measurements. Measurements obtained with these devices during routine or official inspections in pig housing should be interpreted in the context of these inter-device differences, and any exceedance of threshold values should be confirmed using alternative measurement methods. Furthermore, the results of this study demonstrate that various factors significantly influence carbon dioxide and ammonia concentrations within a barn. Therefore, multiple sampling points should be selected within each compartment to adequately capture spatial variability and to determine optimal sensor placement. Although measurement height significantly affected carbon dioxide concentrations, the practical relevance appears limited. In contrast, ammonia concentrations were not influenced by measurement height. As this study was conducted in a single compartment, further research is needed across different barn designs. While measurement outside the animal area may be practical for continuous monitoring, spot checks at animal height are recommended to ensure data reliability.

## Ethics approval and consent to participate

Sampling procedures were in accordance with the German Animal Welfare Act and the regulations on the welfare of animals used for experiments or for other scientific purposes. The experiments did not include any invasive procedure involving the animals. The study design was reviewed and approved by the Animal Welfare Officer of the University of Veterinary Medicine Hannover, Foundation, Hannover, Germany (TV0–2021-V-67).

## Consent for publication

Not applicable.

## Availability of data and materials

The datasets generated during the current study are available from the authors on reasonable request.

## Funding

None.

## CRediT authorship contribution statement

**Thies J. Nicolaisen:** Writing – review & editing, Writing – original draft, Validation, Data curation. **Nina Volkmann:** Writing – review & editing, Investigation, Formal analysis, Data curation. **Lena Brügge:** Investigation, Data curation. **Rolf Nathaus:** Writing – review & editing, Conceptualization. **Nicole Kemper:** Writing – review & editing, Supervision, Conceptualization. **Jochen Schulz:** Writing – review & editing, Supervision, Methodology, Conceptualization.

## Declaration of competing interest

The authors declare that they have no competing interests.

## Appendix Supplementary materials

Image, application 1
