# Supplementary material for: Influence of measurement method, measurement height, and sampling location on carbon dioxide and ammonia concentration in a conventional piglet nursery
Source: Vet Anim Sci. 2026 Aug 14;33:100815. doi: 10.1016/j.vas.2026.100815 (PMC13524847; doi:10.1016/j.vas.2026.100815)
Supplement: Supplementary file 1 [file mmc1.pdf]

Influence of measurement method, measurement height, and sampling location on carbon dioxide and ammonia concentration in a conventional piglet nursery

Nicolaisen et al.

Table S1

Animals (n)	Weight (kg)	Activity factor	CO_2 indoor (ppm)	CO_2 outdoor (ppm)	Temp. (°C)	Air flow (m^3/h)	Air flow per animal (m^3/h)	Date	Measurement period	NH^3 (ppm)
500	13	1.2	2896	420	29.7	3159	6.3	21.01.2021	13:03-13:26	32
498	16.7	1.2	3146	420	30.8	3357	6.7	28.01.2021	12:17-12:48	40
496	17.2	1.2	2854	420	28.6	3938	7.9	02.02.2021	17:02-17:17	36
494	20	1.2	4621	420	28.5	2525	5.1	10.02.2021	12:17-12:36	50
492	24	1.2	4450	420	28.7	2959	6.0	17.02.2021	08:40-08:59	28
500	7.8	1.2	4242	420	30.1	1410	2.8	25.02.2021	11:47-12:00	21
498	12.2	1.2	4675	420	28.6	1776	3.6	04.03.2021	10:44-11:04	31
496	16.1	1.2	4058	420	26.6	2583	5.2	11.03.2021	11:11-11:28	25
494	18.2	1.2	3212	420	31.2	3432	6.9	18.03.2021	11:15-11:29	33
492	20.4	1.2	2567	420	30.9	4828	9.8	23.03.2021	08:45-09:01	36
490	24.7	1.2	3304	420	28.7	4197	8.6	01.04.2021	10:40-10:55	30
487	28.3	1.2	4358	420	28.5	3352	6.9	10.04.2021	10:07-10:21	18
486	33	1.2	4462	420	28.3	3610	7.4	15.04.2021	11:34-11:49	18
500	8.3	1.2	4233	420	28.3	1515	3.0	22.04.2021	11:42-11:50	19
498	12.9	1.2	3320	420	29.8	2668	5.4	29.04.2021	12:20-12:35	16
496	14.9	1.2	3016	420	29.3	3308	6.7	06.05.2021	11:50-12:01	42
494	19.4	1.2	2950	420	27.2	4177	8.5	12.05.2021	11:41-11:54	29
492	24.9	1.2	2492	420	31	5716	11.6	20.05.2021	13:26-13:38	42
490	29.9	1.2	2833	420	29.7	5614	11.5	27.05.2021	10:32-10:43	52
488	34.7	1.2	2137	420	29.1	8715	17.9	02.06.2021	10:35-10:49	24
486	36.5	1.2	1883	420	29.5	10458	21.5	10.06.2021	10:18-10:34	21
500	7.9	1.2	1779	420	29.5	4036	8.1	17.06.2021	11:41-11:53	17
498	11.1	1.2	3066	420	32.3	2538	5.1	24.06.2021	10:42-11:04	18
496	15.8	1.2	2588	420	29	4145	8.4	01.07.2021	11:24-11:50	30
494	20.3	1.2	1762	420	28.8	7953	16.1	08.07.2021	10:33-10:45	25

492	28.1	1.2	2038	420	31.5	7875	16.0	15.07.2021	10:53-11:04	39
488	32.3	1.2	2933	420	30.5	5582	11.4	29.07.2021	11:30-11:41	17
486	34.1	1.2	2575	420	29.7	6784	14.0	03.08.2021	10:20-10:33	14
484	35.3	1.2	2900	420	29.1	6049	12.5	10.08.2021	09:05-09:16	14
484	36.2	1.2	2746	420	29.6	6508	13.4	12.08.2021	11:00-11:12	12
